# Klinisch-praktische Aspekte der Vorbereitung, Durchführung und Nachbetreuung beim Schwangerschaftsabbruch im ersten Trimenon

**DOI:** 10.1007/s00103-024-03981-8

**Published:** 2024-12-03

**Authors:** Matthias David

**Affiliations:** Campus Virchow-Klinikum, Klinik für Gynäkologie, Charité – Universitätsmedizin Berlin, Augustenburger Platz 1, 13353 Berlin, Deutschland

**Keywords:** Schwangerschaftsabbruch, Erstes Trimenon, Medikamentöse Abruptio, Operative Abruptio, Präoperative Maßnahmen, Abortion, First trimester, Medical abortion, Surgical abortion, Preoperative measures

## Abstract

Im Januar 2023 erschien die „S2k-Leitlinie zum Schwangerschaftsabbruch im ersten Trimenon“ der Arbeitsgemeinschaft der Wissenschaftlichen Medizinischen Fachgesellschaften e. V. (AWMF), die vom Bundesministerium für Gesundheit (BMG) beauftragt wurde. Die Leitlinie befasst sich mit dem operativen und dem medikamentösen Schwangerschaftsabbruch nach Beratungsregelung, mit kriminologischer oder mit medizinischer Indikation in den ersten 14 Schwangerschaftswochen, bezogen auf die letzte Regelblutung. In diesem Übersichtsartikel werden, orientiert an dieser Leitlinie, die Abläufe beim medikamentösen und operativen Schwangerschaftsabbruch vor- sowie Vor- und Nachteile der beiden Methoden gegenübergestellt. Klinisch-praktische Aspekte der Vorbereitung und Durchführung, des Komplikationsmanagements und der unmittelbaren Nachbetreuung beim Schwangerschaftsabbruch werden präsentiert.

## Abstract (en)

In January 2023, the “S2k guideline on abortion in the first trimester” of the Association of Scientific Medical Societies (“Arbeitsgemeinschaft der Wissenschaftlichen Medizinischen Fachgesellschaften e. V.”, AWMF) was published, which was commissioned by the Federal Ministry of Health (“Bundesministerium für Gesundheit”, BMG). The guideline deals with surgical and medical abortion after counseling, with criminological or medical indication in the first 14 weeks of pregnancy, based to the last menstrual period. In this review article, based on this guideline, the procedures for medical and surgical abortion are compared as well as the advantages and disadvantages of the two methods. Clinical and practical aspects of preparation and implementation, complication management, and immediate aftercare for abortion are presented.

## Einleitung

In der Bundesrepublik Deutschland wird die ärztliche Weiterbildung durch die sog. (Muster‑)Weiterbildungsordnung der Bundesärztekammer geregelt. In den einzelnen Bundesländern bzw. den entsprechenden Ärztekammern werden dann an diese angelehnte Weiterbildungsordnungen beschlossen, die in regelmäßigen Abständen novelliert werden. In der Fassung der aktuellen (Muster‑)Weiterbildungsordnung 2018 vom 29.06.2023 wird die Durchführung von Schwangerschaftsabbrüchen nicht als Weiterbildungsinhalt für angehende Fachärztinnen und -ärzte genannt, wohl aber die „Beratung bei Schwangerschaftskonflikten sowie Indikationsstellung zum Schwangerschaftsabbruch unter Berücksichtigung gesundheitlicher einschließlich psychischer Risiken“ unter der Rubrik „Handlungskompetenz – Erfahrungen und Fertigkeiten“ im Fachgebiet Frauenheilkunde und Geburtshilfe [1]. Niemand ist verpflichtet, an einem Schwangerschaftsabbruch mitzuwirken. Eine Ausnahme stellen nur Situationen dar, in denen ärztliche Behandlung und Betreuung erforderlich sind, „… um von der Frau eine anders nicht abwendbare Gefahr des Todes oder einer schweren Gesundheitsschädigung abzuwenden …“ [2]. „Auch zur Mitwirkung an einem Schwangerschaftsabbruch im Rahmen der ärztlichen Weiterbildung oder der Teilnahme an entsprechenden Fortbildungsmaßnahmen kann ein Arzt/eine Ärztin nicht verpflichtet werden …“ [3].

Beim Schwangerschaftsabbruch handelt es sich um eine in der modernen Medizin einzigartige Maßnahme, die mit keiner anderen ärztlichen Handlung zu vergleichen ist, auch wenn vom „technischen Ablauf“ her das operative Vorgehen mittels Saugkürettage bei den verschiedenen Fehlgeburtsformen dem Prozedere bei einem Schwangerschaftsabbruch gleicht. Es gibt eine Reihe von Pro- und Kontraargumenten bzgl. eines Schwangerschaftsabbruchs aus Sicht der betroffenen Frauen, aus ethisch-moralischer und aus ärztlicher Perspektive (siehe [4]), die nachvollziehbar machen, warum einige Frauenärztinnen und -ärzte Schwangerschaftsabbrüche durchführen und andere nicht. Dieses verbriefte Weigerungsrecht ist eine wichtige Errungenschaft, die letztlich auch auf die bundesdeutsche Kompromissneuregelung des § 218 nach der deutschen Wiedervereinigung vor nahezu 30 Jahren zurückgeht [5]. Auf ethische Implikationen und die rechtlichen Regelungen wird an dieser Stelle nicht weiter eingegangen, sondern dafür auf andere Beiträge in diesem Themenheft verwiesen.

Das Bundesministerium für Gesundheit (BMG) hat vor einigen Jahren die Deutsche Gesellschaft für Gynäkologie und Geburtshilfe (DGGG) mit der Erstellung einer Leitlinie zum „Schwangerschaftsabbruch im ersten Trimenon“ beauftragt, die schließlich im Januar 2023 der (Fach‑)Öffentlichkeit vorgestellt wurde^1^ [6]. Wie diese Leitlinie befassen sich die nachfolgenden Ausführungen ausschließlich mit dem operativen und dem medikamentösen Schwangerschaftsabbruch nach Beratungsregelung, mit kriminologischer oder mit medizinischer Indikation in den ersten 14 Schwangerschaftswochen (SSW) bezogen auf die letzte Regelblutung (= post menstruationem, p. m.). Ziel dieses Beitrages ist es, klinisch-praktische Aspekte der Vorbereitung, Durchführung und unmittelbaren Nachbetreuung beim Schwangerschaftsabbruch vorzustellen. Die Darstellung folgt inhaltlich und in der Systematik der o. g., 2023 veröffentlichten S2k-Leitlinie „Schwangerschaftsabbruch im ersten Trimenon“ [3].

## Maßnahmen vor dem Schwangerschaftsabbruch

### Feststellung der Schwangerschaft

Viele Frauen führen bei Ausbleiben der Regelblutung bereits zu Hause einen Schwangerschaftstest durch, bevor sie eine Praxis oder Klinik aufsuchen. Hierbei handelt es sich um einen qualitativen Urintest, der das Schwangerschaftshormon β‑hCG nachweist. Die Vorstellung in einer Arztpraxis oder einer Frauenklinik dient dann häufig der Bestätigung des positiven Testergebnisses. Eine Entscheidung, ob die Schwangerschaft ausgetragen werden soll oder nicht, ist dann seitens der betroffenen Frau häufig schon zuvor gefallen. Ergänzend zur β‑hCG-Bestimmung im Urin der Frau wird eine vaginale Ultraschalluntersuchung durchgeführt, um den Sitz, das Alter und die Vitalität der Schwangerschaft festzustellen.

Als sichere sonografische Schwangerschaftszeichen gelten der Nachweis einer Fruchthöhle, eines darin befindlichen Dottersackes oder eines Embryos mit Herzaktion im Uterus. „Eine sich normal intrauterin entwickelnde Schwangerschaft kann mit hochauflösenden Vaginalsonden frühestens ab dem 32. bis 36. Tag post menstruationem (p. m.), also kurz nach Ausbleiben der Regelblutung, in Form einer wenige Millimeter großen, exzentrisch gelegenen Chorionhöhle sonografisch dargestellt werden. … Embryonale Herzaktionen können ab 5 + 5 SSW p. m. nachweisbar sein“ [3]. Trotz der aktuell vorhandenen apparativen (Ultraschall) und labortechnischen (schnell verfügbarer Schwangerschaftstest, serielle β‑hCG-Bestimmungen) Möglichkeiten kann es schwierig sein, eine (u. U. noch nicht sonografisch nachweisebare) intrauterine von einer (mglw. noch nicht sichtbaren) extrauterinen bzw. ektopen Schwangerschaft abzugrenzen. Solange dies nicht gelingt, spricht man von einer „Schwangerschaft unbekannter Lokalisation“. Diesen Patientinnen wird dann eine quantitative β‑hCG-Bestimmung im Serum und eine Kontrollultraschalluntersuchung alle 2 bis 3 Tage empfohlen, bis der intrauterine Sitz der Gravidität sicher bewiesen ist [7]. Erst dann erscheint es sinnvoll, den Schwangerschaftsabbruch zu planen. In der AWMF-S2k-Leitlinie zum Schwangerschaftsabbruch im ersten Trimenon wird allerdings auch auf ein alternatives Vorgehen hingewiesen: Demnach „kann bei beschwerdefreien Frauen nach ausführlicher Aufklärung zur Vermeidung eines belastenden Abwartens ein medikamentöser Abbruch durchgeführt werden“, auch ohne dass der intrauterine Schwangerschaftssitz sicher festgestellt wurde. Der Patientin sind nachfolgend Verlaufskontrollen des β‑hCG-Wertes zu empfehlen und sie soll über das geringe Restrisiko einer weiter bestehenden ektopen Gravidität informiert werden [3].

### Information und Aufklärung

Die Frauen, die einen Schwangerschaftsabbruch in Erwägung ziehen, sollen frühzeitig und einfühlsam über die verschiedenen rechtlichen, sozialen und medizinischen Aspekte des Schwangerschaftsabbruchs informiert werden. Dieses ärztliche Beratungsgespräch ist nicht gleichzusetzen mit dem Gespräch im Rahmen der Beratungspflicht gemäß Schwangerenkonfliktgesetz. Hier heißt es im Abschnitt 2, Paragraph 5: „Die nach § 219 des Strafgesetzbuches notwendige Beratung ist ergebnisoffen zu führen. Sie geht von der Verantwortung der Frau aus. Die Beratung soll ermutigen und Verständnis wecken, nicht belehren oder bevormunden. Die Schwangerschaftskonfliktberatung dient dem Schutz des ungeborenen Lebens …“ [2].

Entsprechend der AWMF-Leitlinie zum Schwangerschaftsabbruch im ersten Trimenon sind Vorerkrankungen und psychosoziale Rahmenbedingungen bei der Darstellung der beiden Optionen medikamentöser und operativer Schwangerschaftsabbruch und der sich daraus ergebenden individuellen Risikoaufklärung zu berücksichtigen. Nur so kann der Schwangeren eine informierte Entscheidung ermöglicht werden. „Die Methode des Schwangerschaftsabbruchs, die Art der Anästhesie, der Ort der Durchführung, die organisatorischen Abläufe, die Nachuntersuchung sind für die Schwangere der konkreten Situation entsprechend anzupassen …“ [3].

Schwangerschaftsabbrüche sind sichere medizinische Maßnahmen. Nur sehr selten treten schwere Komplikationen wie starke Blutungen, eine Uterusperforation oder eine schwere Entzündung bis hin zur Sepsis auf, wobei das Komplikationsrisiko mit dem Schwangerschaftsalter, in dem der Abbruch durchgeführt wird, etwas zunimmt [8]. Die Tab. 1 stellt die möglichen Komplikationen und Risiken von medikamentösem und operativem Schwangerschaftsabbruch gegenüber. In der wissenschaftlichen Literatur lassen sich keine Beweise dafür finden, dass insgesamt eine der beiden Methoden riskanter ist als die andere [9].

**Tab. 1:** Vergleich der Risiken beim medikamentösen und operativen Schwangerschaftsabbruch (aus [3], modifiziert)

	Medikamentöser Schwangerschaftsabbruch	Operativer Schwangerschaftsabbruch
Starke Blutungen	10 von 1000	2 von 1000
Weiterbestehende Schwangerschaft	10 von 1000	2 von 1000
(Erneute) Vakuumaspiration/Absaugung wegen Geweberesten	30 bis 50 von 1000	30 von 1000
Infektionen	1 bis 2 von 1000	0 bis 110 von 1000; mit Antibiotikaprophylaxe < 20 von 1000
Uterusperforation	(Entfällt methodenbedingt)	1 von 1000
Zervixverletzung	(Entfällt methodenbedingt)	1 bis 6 von 1000, mit Zervixpriming geringer
Narkosekomplikationen	(Entfällt methodenbedingt)	2 von 1000
Fertilitätsstörung, Abort, ektope Schwangerschaft	Risiko nicht erhöht	Risiko nicht erhöht
Plazentationsstörungen: Plazenta praevia	Risiko nicht erhöht	Risiko erhöht bei Verwendung von Metallküretten, nicht bei Vakuumaspiration
Frühgeburtlichkeit	Risiko nicht erhöht	Widersprüchliche Daten; bei Schwangerschaften nach dem Jahr 2000 wahrscheinlich nicht erhöht

Die Frauen werden auch darüber informiert, beim Auftreten welcher Symptome sie unmittelbar eine ärztliche Praxis oder eine Klinik aufsuchen sollten. Dabei wird auch dargestellt, welche Beschwerden als Nach- und Nebenwirkungen des medikamentösen bzw. operativen Schwangerschaftsabbruchs zu erwarten und als „normal“ einzustufen sind (insbes. moderate, teils krampfartige Unterbauchschmerzen, Blutungen, gastrointestinale Symptome, Nebenwirkungen der Narkose [3]).

Im Zuge des Gesprächs über mögliche Komplikationen muss auch thematisiert werden, dass in sehr seltenen Fällen der Abbruch fehlschlagen und die Schwangerschaft fortbestehen kann oder dass zumindest relevante Schwangerschaftsgewebereste im Uteruskorpus zurückbleiben, die weitere Maßnahmen, z. B. einen (erneuten) operativen Eingriff, erfordern.

Zur Frage, ob sich ein Schwangerschaftsabbruch negativ auf die Umsetzung eines späteren Kinderwunsches auswirkt, heißt es in der Leitlinie zum Schwangerschaftsabbruch im ersten Trimenon: „Medikamentöse und Schwangerschaftsabbrüche mittels Vakuumaspiration haben wahrscheinlich keine Auswirkungen auf die spätere Fertilität, die Häufigkeit von Spontanaborten sowie ektope Graviditäten. Die Evidenz ist begrenzt auf ältere Studien mit methodischen Schwächen … Das Risiko von Frühgeburten ist nach medikamentösen Abbrüchen offenbar nicht erhöht. Zu operativen Abbrüchen im ersten Trimenon sind die Daten widersprüchlich …“ [3].

Die dargestellten medizinischen Informationen werden zur Entscheidungsfindung der Schwangeren beitragen und diese unterstützen. In der schon zitierten S2k-Leitlinie zum Schwangerschaftsabbruch wird betont, dass Ärztinnen und Ärzte mit ihrer Erfahrung und Kompetenz zwar am Entscheidungsfindungsprozess beteiligt sind, es handelt sich aber dennoch um keine gemeinsame Entscheidung. Diese trifft die Schwangere letztlich allein entsprechend ihren persönlichen Präferenzen, Lebensumständen, Werten und Überzeugungen [3].

## Medikamentöser Schwangerschaftsabbruch

### Ort und Art der Durchführung

Ein Schwangerschaftsabbruch kann medikamentös oder operativ durchgeführt werden. Insgesamt wird die Effektivität eines medikamentösen Abbruchs mit ca. 95 % angegeben, wobei diese umso höher ist, je früher die Medikamentengabe in der Schwangerschaft erfolgt [10, 11]. Ein medikamentöser Abbruch wird in Deutschland fast immer ambulant in einer gynäkologischen Praxis oder in einer gynäkologischen Poliklinik o. Ä. durchgeführt. Die Kombination der Wirkstoffe Mifepriston und Misoprostol ist dafür die am häufigsten angewandte Medikamentenkombination und kann als internationaler Standard beim medikamentösen Schwangerschaftsabbruch gelten [12]. Anders als bei der Durchführung der (Saug‑)Kürettage, dem operativen Schwangerschaftsabbruch, ist hier keine Narkose notwendig. „Allerdings ist die Dauer der Behandlung bei medikamentöser Abortinduktion vergleichsweise länger, es kommt häufiger zu Blutungen und Bauchkrämpfen und es sind mehrere ärztliche Kontrolltermine erforderlich …“ ([13]; Tab. 2).

**Tab. 2:** Vor- und Nachteile eines medikamentösen gegenüber einem operativen Schwangerschaftsabbruch (nach [8], modifiziert und ergänzt)

Medikamentöser Schwangerschaftsabbruch	Operativer Schwangerschaftsabbruch
Vermeidung invasiver/operativer Maßnahmen	Immer invasive Maßnahme
Ähnelt einer Fehlgeburt	Gleiches Vorgehen wie bei Abortkürettage
Kann zu Hause stattfinden	Anästhesie nötig, meist sog. Vollnarkose
Bis zur vollständigen Beendigung der Schwangerschaft können Tage bis Wochen vergehen	Findet immer in einer medizinischen Einrichtung statt, meist ambulant
Ist mit krampfartigen Unterbauchschmerzen und u. U. stärkeren Blutungen verbunden	Erfordert mindestens 2 Arztbesuche
Verwendete Medikamente können Übelkeit, Erbrechen, Durchfall, Schüttelfrost und Fieber verursachen	Die zum Zervixpriming verwendeten Medikamente verursachen Krämpfe und Blutungen und können Übelkeit, Erbrechen, Durchfall, Schüttelfrost und Fieber nach sich ziehen
Schwerwiegende Komplikationen sind selten	Ist in einem vorhersehbaren Zeitraum abgeschlossen
Keine Anästhesie erforderlich	Nachsorge nicht zwingend erforderlich
Erfordert 2 oder mehr Arztbesuche	Meist 2‑stufiger Prozess
Nachkontrolle, um Vollständigkeit des Schwangerschaftsabgangs zu bestätigen	Schwerwiegende Komplikationen sind selten
Immer ein mehrstufiger Prozess	Nach dem Eingriff 1–2 Wochen lang leichte Schmerzen und Blutungen

Mifepriston ist ein Progesteron-Rezeptor-Antagonist, der sich als Antigestagen kompetitiv an Progesteronrezeptoren bindet und eine Deziduanekrose, eine Erweichung des Zervixgewebes, vermehrte Uteruskontraktilität sowie eine erhöhte Prostaglandinsensitivität nach sich zieht. Mifepriston allein ist bei einem medikamentösen Schwangerschaftsabbruch nur begrenzt, in Kombination mit Misoprostol jedoch hoch wirksam [12]. Meist wird Mifepriston 1–2 Tage vor Misoprostol appliziert. Bei Misoprostol handelt es sich um ein Prostaglandin-E1-Analogon, welches zu Uteruskontraktionen und einer Zervixeröffnung führt [13].

In der für Deutschland gültigen Fachinformation für Mifepriston findet sich folgender Passus: „Zum Abbruch einer Schwangerschaft dürfen Mifegyne 600® und das Prostaglandin nur in Übereinstimmung mit den aktuell gültigen Gesetzen und Bestimmungen verschrieben und verabreicht werden … Diese Arzneimittel müssen unter Aufsicht eines Arztes oder von diesem ermächtigtes medizinisches Fachpersonal angewendet werden“ [14].

Laut Herstellerangaben ist das Mifepriston-Präparat in Deutschland für die Durchführung eines medikamentösen Abbruchs einer frühen intrauterinen Schwangerschaft bis zum 63. Tag der Amenorrhö bei anschließender Applikation eines Prostaglandinanalogons zugelassen. Die Art der Anwendung unterscheidet sich je nach Schwangerschaftsdauer (bis zum 49. Tag nach letzter Regel vs. 50. bis 63. Tag der ausbleibenden Blutung): (1) Bis zum 49. Tag (Ende der 7. Schwangerschaftswoche) soll laut Zulassung einmalig oral 600 mg Mifepriston eingenommen werden und 36–48 h danach 400 µg Misoprostol. (2) Vom 50. bis 63. Tag (Anfang der 8. bis Ende der 9. Schwangerschaftswoche) soll 36–48 h nach der Einnahme von 600 mg Mifepriston laut Zulassung das Prostaglandinanalogon Gemeprost (1 mg) vaginal verabreicht werden [14].

Dieses Prozedere gilt für den medikamentösen Schwangerschaftsabbruch bei erwachsenen Frauen; für die Anwendung von Misoprostol bei Jugendlichen liegen nur begrenzte Erfahrungen vor. Laut Zulassung des Medikaments für Deutschland ist ausschließlich die oben dargestellte orale Applikation vorgesehen, Misoprostol kann prinzipiell aber auch sublingual, bukkal oder intravaginal verabreicht werden. Applikation und Dosierung der beiden Medikamente sind beim medikamentösen Schwangerschaftsabbruch international nicht einheitlich. Sowohl die o. g. unterschiedlichen Applikationsformen als auch modifizierte Dosierungen wurden in Studien untersucht und werden in anderen Ländern auch entsprechend den dort geltenden Leitlinien verabreicht [15–17]. Vaginal appliziertes Misoprostol ist wirksamer als oral verabreichtes und hat weniger gastrointestinale Nebenwirkungen als bei sublingualer oder bukkaler Gabe [10]. Jede Abweichung von der Zulassung, z. B. die Anwendung der Kombination Mifepriston plus Misoprostol ab Beginn der 8. Schwangerschaftswoche, ist als „off label use“ anzusehen. „Unter Off-Label-Use wird der zulassungsüberschreitende Einsatz eines Arzneimittels außerhalb der von den nationalen oder europäischen Zulassungsbehörden genehmigten Anwendungsgebiete (Indikationen, Patientengruppen) verstanden. Grundsätzlich ist Ärztinnen und Ärzten eine zulassungsüberschreitende Anwendung von Arzneimitteln erlaubt. Eine umfassende gründliche Aufklärung der Patientinnen und Patienten zum möglichen Nutzen und zu möglichen Risiken des Off-Label-Use, deren Zustimmung zum Einsatz des Medikamentes und eine lückenlose Behandlungsdokumentation durch die Ärztin oder den Arzt sind in jedem Fall unerlässlich“ [18].

### Umgang mit Nebenwirkungen

Für den Umgang mit Blutungen, Schmerzen oder gastrointestinalen Nebenwirkungen im Rahmen des medikamentösen Schwangerschaftsabbruches ist es wichtig, die betroffenen Frauen darüber zu informieren, was typischerweise an Symptomen zu erwarten ist. Auch darüber, wie das abgehende Schwangerschaftsgewebe aussieht, und dass es ab der 8./9. Schwangerschaftswoche möglich ist, einen Embryo zu erkennen, sollte gesprochen werden. In der Leitlinie zum Schwangerschaftsabbruch im ersten Trimenon wird ausgeführt, dass nach Anwendung des Uteruskontraktionen auslösenden Prostaglandinpräparates „die Anwesenheit einer vertrauten Person zur Unterstützung sinnvoll und die ständige (‚24/7‘) telefonische Erreichbarkeit eines Arztes/einer Ärztin für Fragen und Notfälle unabdingbar …“ ist [3]. Nach der Einnahme von Mifepriston treten normalerweise keine, nach der Misoprostol-Gabe dagegen häufig Nebenwirkungen auf. Dazu gehören 4–6 h andauernde starke uterine Krämpfe und vaginale Blutungen, die stärker sind als eine typische Menstruationsblutung und oft den Abgang von Blutgerinnseln und des Schwangerschaftsgewebes umfassen. Nach den ersten 4–6 h nach der Prostaglandinapplikation lässt die Blutung allmählich nach; Schmierblutungen treten danach häufig noch für 1–2 Wochen auf [12]. Für ein adäquates Schmerzmanagement wird die rechtzeitige Einnahme nichtsteroidaler Antiphlogistika empfohlen und wegen des häufigen Auftretens von Übelkeit und Erbrechen bei der Anwendung von Misoprostol auch ein Antiemetikum.

Beim medikamentösen Schwangerschaftsabbruch kann bei 0,5–1 % der Frauen die Schwangerschaft weiterbestehen und bei 3–5 % verbleiben Reste [19, 20]. Liegt der Verdacht auf solche Residuen vor, sind also Embryo, Fruchthöhle und Plazenta nicht vollständig abgegangen, so gibt es laut AWMF-Leitlinienempfehlung 3 Therapieoptionen, die der betroffenen Frau dargelegt werden sollten: (1) Abwarten der nächsten Regelblutung, es sei denn, es kommt zwischenzeitlich zu verstärkten Blutungen aus dem Uterus oder bei Infektionszeichen (Endomyometritis); (2) Gabe eines Prostaglandinpräparates wie Misoprostol; (3) operative Absaugung des Uterusinhalts in Narkose und möglichst mit perioperativer Ultraschallkontrolle [3].

Während ein inkompletter Abgang des Schwangerschaftsgewebes sich meist durch Blutungen und Schmerzen bemerkbar macht, kann eine weiterbestehende Schwangerschaft ohne eine Nachkontrolle unbemerkt bleiben. Deshalb ist eine Ultraschalluntersuchung in einem geeigneten Abstand zur Medikamentenapplikation, z. B. 2 Wochen nach der Blutungsauslösung, zur Bestätigung eines vollständigen Schwangerschaftsabbruchs sinnvoll [3].

## Operativer Schwangerschaftsabbruch

### Präoperative Maßnahmen

Bei Frauen mit einem erhöhten Operations- und/oder Narkoserisiko, die sich für einen operativen Schwangerschaftsabbruch entschieden haben, sind u. U. zusätzliche Untersuchungen nötig, auch um einschätzen zu können, ob eine ambulante Durchführung des Eingriffs möglich ist oder ob die Operation besser stationär in einer Klinik erfolgen sollte. Bei allen Frauen erfolgt eine ausführliche ärztliche Operations- und eine Narkoseaufklärung. „Die Wahl des Anästhesieverfahrens unter Berücksichtigung der Risikokonstellation und der Präferenz der Patientin sowie der erforderlichen operativen Bedingungen obliegt dem anästhesiologischen Personal. Grundsätzlich zählt der operative Schwangerschaftsabbruch zu den kleinen gynäkologischen Eingriffen mit kurzer Eingriffsdauer. Für das ambulante Setting ist vorwiegend die Allgemeinanästhesie als total intravenöse Anästhesie (TIVA) mit kurzwirksamen Opioiden geeignet.“ [3].

Teil der präoperativen Aufklärung ist auch die Information über das sog. Zervixpriming. Mit einer Zervixreifung bzw. Erweichung des Gebärmutterhalses durch ein Prostaglandinpräparat wie Misoprostol wird die erforderliche Kraft, um den Gebärmutterhals für einen operativen Schwangerschaftsabbruch im ersten Trimester aufzudehnen, aber auch das Risiko für einen unvollständigen Schwangerschaftsabbruch reduziert [21]. „Bei vorbekanntem erhöhtem Risiko für operative Komplikationen sollte der Schwangerschaftsabbruch nur in Einrichtungen erfolgen, die strukturell und personell in der Lage sind, diese bei deren Eintritt unverzüglich adäquat behandeln zu können.“ [3].

### Durchführung und Komplikationsmanagement

Operative Schwangerschaftsabbrüche haben unabhängig vom Schwangerschaftsalter eine hohe Erfolgsrate und ein sehr geringes Risiko schwerwiegender Komplikationen von unter 0,1 % [22]. Die Vakuumaspiration und die Ausschabung mit einer stumpfen Metallkürette (= Kürettage) sind sichere Methoden zum Schwangerschaftsabbruch [10]. Die Vakuumaspiration (Syn.: Saugkürettage) ist im ersten Trimenon der Schwangerschaft die operative Methode der Wahl [3]. Eine (scharfe) Metallkürettage wird nicht als Ersatz für die Vakuumaspiration empfohlen, allerdings ist die wissenschaftliche Evidenz für diese Empfehlung gering [23].

Die AWMF-Leitlinie zum Schwangerschaftsabbruch unterscheidet einen operativen Schwangerschaftsabbruch vor und einen nach der 7. Schwangerschaftswoche: Beim sog. frühen Abbruch (unter 7 Schwangerschaftswochen) kann die relativ geringe Gewebemenge, die hier abgesaugt wird, die Entscheidung darüber, ob die Schwangerschaft vollständig entfernt wurde, erschweren. Bei Unsicherheit darüber sollte eine routinemäßige peri- und postoperative Ultraschalluntersuchung, eine direkte Untersuchung des entfernten Schwangerschaftsgewebes und eine β‑hCG-Bestimmung im Verlauf erfolgen [23]. Laut Angaben in einer kanadischen Leitlinie ist das unbeabsichtigte Zurücklassen von Schwangerschaftsgewebe im Uterus nach einer Vakuumaspiration durch einen erfahrenen Arzt bzw. eine erfahrene Ärztin selten und wird in dieser Konstellation mit 0,7–4 % angegeben. Nach erfolgreicher Absaugung der Schwangerschaft wird innerhalb von 24 h ein Abfall des β‑hCG-Wertes im Serum auf die Hälfte des Vorwertes erwartet [23].

Vor Durchführung der Saugkürettage ist fast immer eine (vorsichtige) mechanische Aufdehnung des Zervikalkanals mit Metall- oder Kunststoffstiften notwendig, damit dann eine für die Schwangerschaftswoche ausreichend große Absaugkanüle in das Cavum uteri eingeführt werden kann. Ein Zervixpriming durch die Applikation eines Prostaglandinpräparates (z. B. Misoprostol) oder die Einlage von stäbchenförmigen osmotischen Hydrogeldilatatoren einige Stunden vor der Saugkürettage erleichtert die Aufdehnung des Zervikalkanals und vermindert die Gefahr von Zervixverletzungen. Der Blutverlust im Rahmen eines operativen Schwangerschaftsabbruchs ist in der Regel gering bis moderat. Bei den seltenen stärkeren Blutungen ist die intravenöse Gabe von Oxytocin oder die rektale Applikation von Misoprostol zur Auslösung von Uteruskontraktionen hilfreich. Zu Verhinderung einer Infektion kann eine perioperative Antibiotikaprophylaxe als Einmaldosis vor Beginn der Operation verabreicht werden [3].

Perioperative Komplikationen treten im Rahmen der Vakuumaspiration zum Schwangerschaftsabbruch sehr selten auf. In einer Übersichtsarbeit wurden folgende Häufigkeiten angegeben: Eine erneute Saugkürettage war in ≤ 3,0 % nötig, verstärkte Blutungen, die allerdings keine Transfusion erforderten, wurden in 0–4,7 % der Eingriffe verzeichnet; schwerwiegende Komplikationen, wie z. B. eine Uterusperforation, die einen operativen Eingriff, und Blutungen, die eine Transfusion erforderlich machen, traten bei ≤0,1 % der Patientinnen auf; anästhesiebedingte Komplikationen wurden bei ≤0,5 % der Eingriffe beobachtet [22].

In der AWMF-Leitlinie zum Schwangerschaftsabbruch im ersten Trimenon wird zur Risikoreduzierung eine präoperative vaginale Ultraschalluntersuchung empfohlen, um eine Retroflexio uteri, Fehlbildungen des Uterus und Plazentationsstörungen auszuschließen [3]. Ebenfalls essenziell ist eine Sonografie, wenn nach einem operativen Schwangerschaftsabbruch der Verdacht auf Reste oder das gänzliche Fortbestehen der Schwangerschaft besteht.

### Feingewebliche Untersuchung des Schwangerschaftsgewebes

Nach einem medikamentösen Schwangerschaftsabbruch liegt in den allermeisten Fällen kein Gewebe zur histopathologischen Untersuchung vor. Die Weltgesundheitsorganisation (WHO) und das britische Royal College of Obstetricians and Gynaecologists (RCOG) sehen laut ihren aktuellen Leitlinien allerdings auch keinen zwingenden Grund, das vorhandene, beim operativen Schwangerschaftsabbruch entfernte Gewebe routinemäßig untersuchen zu lassen, denn es fehle der Nachweis eines Nutzens [8, 24]. In der AWMF-Leitlinie zum Schwangerschaftsabbruch im ersten Trimenon werden allerdings mögliche Vorteile einer solchen histologischen Untersuchung aufgeführt: (1) Beweis für den intrauterinen Sitz der Schwangerschaft; (2) Ausschluss von Trophoblasterkrankungen (in den entwickelten Industriestaaten: Prävalenz der Blasenmole ca. 1 pro 591 Schwangerschaften, Prävalenz für gestationsbedingte Trophoblasterkrankungen (GTD) ca. 1 pro 714 Lebendgeburten [25]); (3) weiterführende Untersuchungen bei einem Schwangerschaftsabbruch nach auffälligem embryonalen bzw. fetalen Befund in der Pränataldiagnostik. „Auf die Möglichkeit eines falsch-positiven Befundes der Pränataldiagnostik sollte bereits bei der Entscheidung zum Schwangerschaftsabbruch, aber auch nochmals vor der weiterführenden Untersuchung des Schwangerschaftsgewebes hingewiesen werden …“ [3].

## Schlussbemerkung

Abschließend sei aus der im Mai 2024 im *Frauenarzt* veröffentlichten gemeinsamen „Stellungnahme zum Schwangerschaftsabbruch in Deutschland. Die Würde aller Beteiligten achten“ der Deutschen Gesellschaft für Gynäkologie und Geburtshilfe und des Berufsverbandes der Frauenärzte zitiert [26]. Diese ist eine kritische Reaktion auf die im April 2024 veröffentlichten Empfehlungen der von der Bundesregierung eingesetzten Arbeitsgruppe 1 der „Kommission zur reproduktiven Selbstbestimmung und Fortpflanzungsmedizin“ [27].

„Jeder Schwangerschaftskonflikt und möglicher Schwangerschaftsabbruch stellen zunächst eine starke Belastung sowohl für die betreffende Frau als auch für das Fachpersonal dar, das an Beratung und Durchführung beteiligt ist. … Die beste Maßnahme zur Reduktion von Schwangerschaftsabbrüchen ist die Prävention von ungeplanten und ungewollten Schwangerschaften. Es ist gesellschaftliche Aufgabe, Rahmenbedingungen zu schaffen, unter denen Frauen Kinder bekommen und großziehen möchten und auch können …“ [26].

## Footnotes

1 Siehe: https://register.awmf.org/de/leitlinie/detail/015-094 (Zugegriffen 23.08.2024).
